# Visceral Leishmaniasis in the Muzaffapur Demographic Surveillance Site: A Spatiotemporal Analysis

**DOI:** 10.4269/ajtmh.18-0448

**Published:** 2018-10-08

**Authors:** Epco Hasker, Paritosh Malaviya, Kristien Cloots, Albert Picado, Om Prakash Singh, Sangeeta Kansal, Marleen Boelaert, Shyam Sundar

**Affiliations:** 1 Department of Public Health, Institute of Tropical Medicine, Antwerp, Belgium;; 2 Institute of Medical Sciences, Banaras Hindu University, Varanasi, India;; 3 Foundation for Innovative New Diagnostics (FIND), Geneva, Switzerland;; 4 ISGlobal, Hospital Clínic, Universitat de Barcelona, Barcelona, Spain

## Abstract

In the Indian subcontinent, visceral leishmaniasis (VL) has a strongly clustered distribution. The “index case approach” is promoted both for active case finding and indoor residual spraying (IRS). Uncertainty exists about the optimal radius. Buffer zones of 50–75 m around incident cases have been suggested for active case finding, for IRS the recommendation is to cover a radius of 500 m. Our aim was to establish optimal target areas both for IRS and for (re)active case finding. We plotted incident VL cases on a map per 6-month period (January–June or July–December) and drew buffers of 0 (same household), 50, 75, 100, 200, 300, 400, and 500 m around these cases. We then recorded total population and numbers of VL cases diagnosed over the next 6-month period in each of these buffers and beyond. We calculated incidence rate ratios (IRRs) using the population at more than 500 m from any case as reference category. There was a very strong degree of spatial clustering of VL with IRRs ranging from 45.2 (23.8–85.6) for those living in the same households to 14.6 (10.1–21.2) for those living within 75 m of a case diagnosed, during the previous period. Up to 500 m the IRR was still five times higher than that of the reference category. Our findings corroborate the rationale of screening not just household contacts but also those living within a perimeter of 50–75 m from an index case. For IRS, covering a perimeter of 500 m, appears to be a rational choice.

## Introduction

Visceral leishmaniasis (VL) is a slowly progressive infectious disease caused—on the Indian subcontinent—by the parasite *Leishmania donovani*. The vector in this region is the sand fly *Phlebotomus argentipes*, which is assumed to have a short flight range.^1^ Humans are considered to be the only reservoir, and the estimated incubation period is 2–6 months.^2^ In the Indian subcontinent, VL is a disease of impoverished rural communities and is known to have a strongly clustered distribution.^3–6^ Clustering is observed in particular at the level of the hamlet and around previous VL cases.^7^ The disease is also strongly associated with poverty and the spatial clustering is probably to a large extent explained by the habitat of communities living in precarious conditions.^8,9^

The main disease control measures are early case detection and treatment and indoor residual spraying (IRS). For active case finding there is no consensus on the size of the target population. Huda et al. applied a radius of 50–75 m around the home of VL patients in their “index-case” strategy, Hirve et al. screened at least 50 consecutive households around index cases diagnosed the previous year, whereas Singh et al. screened all households within a 50 m radius up to a maximum of 100.^10–12^

The guidelines for IRS are not unanimous either. In endemic situations, the World Health Organization recommends deploying IRS in affected villages just before the months with maximum sand fly density. During epidemics on the other hand, large-scale IRS is recommended, covering all buildings, including houses and animal shelters, both in the affected and surrounding areas.^13^ However, they provide no range for defining these “surrounding areas.” Although the 2017 guidelines of the National Vector borne Disease Control program in India have similar twice yearly recommendations of IRS in VL-endemic villages, in addition they state that “Focal spray in the 500 m range of an index case of kala-azar (VL) will be undertaken as soon as a case is reported.”^14^

Most of this guidance is empirical; there are no published studies comparing the relative efficiency of varying perimeters for these targeted interventions. They all share the element of “reactive intervention,” that is interventions implemented in response to a detected VL case, but without proper justification for the size of the target population around that index case that should be covered. Within the VL elimination initiative in the Indian subcontinent, countries have reached or are closing in on the elimination threshold and it becomes increasingly important to target interventions in the most efficient way, as budgetary constraints do increase during the maintenance phase of such elimination efforts. In this study, we used data on VL incidence obtained in a longitudinal population surveillance site in a VL-endemic region in Bihar, India, to study the degree of clustering around VL index cases with the aim of optimizing the targeted strategies. There are substantial and highly variable delays between the date a person is infected with *L. donovani* and the date (s)he is diagnosed with VL. Unfortunately under routine conditions we will never know when a person was infected and by the time (s)he is diagnosed, the onset of symptoms too may be some time in the past. The only information readily available is the date VL cases were diagnosed, to which the exact location can be added. From the perspective of a disease control program, this is the information that can be acted on, either for (re)active case finding or for IRS. Our objectives were to assess whether VL cases diagnosed during the previous 6-month period (January–June or July–December) can be used as “index cases” to target IRS or active case finding interventions and more specifically to establish the most appropriate buffer diameters around such index cases.

## Methods

### Study site.

**Figure 1.**
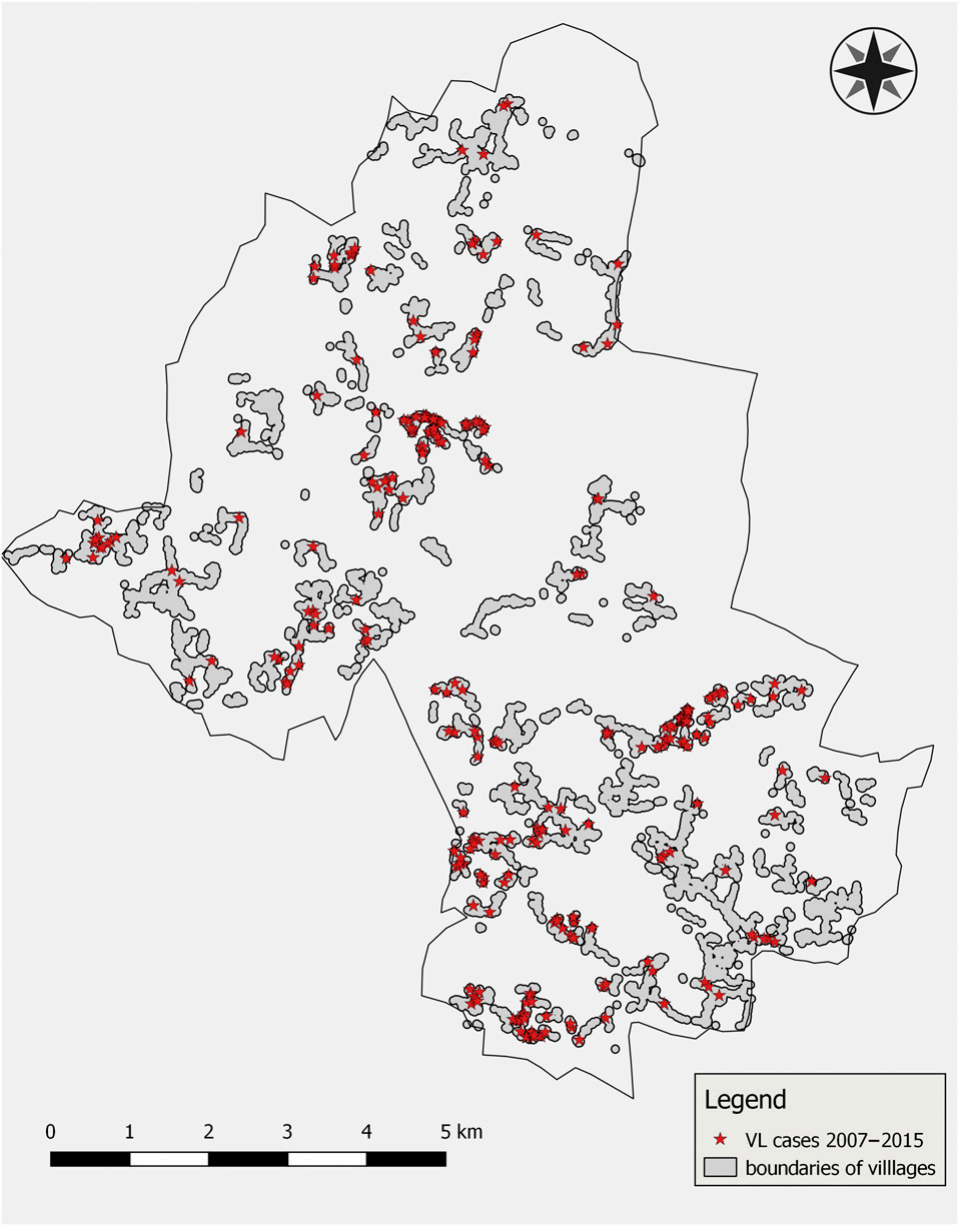
Map of the Muzaffarpur Health and Demographic Surveillance Site showing village boundaries and residence of 328 visceral leishmaniasis (VL) cases observed over the period 2007–2015 (map created in QGIS version 2.14.19-Essen; QGIS Geographic Information System, Open Source Geospatial Foundation, Chicago, IL). This figure appears in color at www.ajtmh.org.

The Muzaffarpur Health and Demographic Surveillance Site (HDSS) is a rural area of Muzaffarpur district, Bihar, India. This geographically continuous area comprises 50 villages with a total population of approximately 90,000 and has been under surveillance since 2008.^15^ Geographic coordinates have been recorded for all households in the area. The area is endemic for VL and since 2008 annual exhaustive household surveys are being conducted. During these surveys, demographic records are updated, and all incident cases of VL since the previous survey are recorded and verified. The first survey recorded VL retrospectively until January 1, 2007. Data are archived in a dataset including observations on 91,908 persons divided over 14,376 households. We exploited the data from the HDSS for the period 2007–2015. In this period 328 new VL cases were reported in this population. Figure 1 shows the distribution of these cases and the village boundaries.

**Figure 2.**
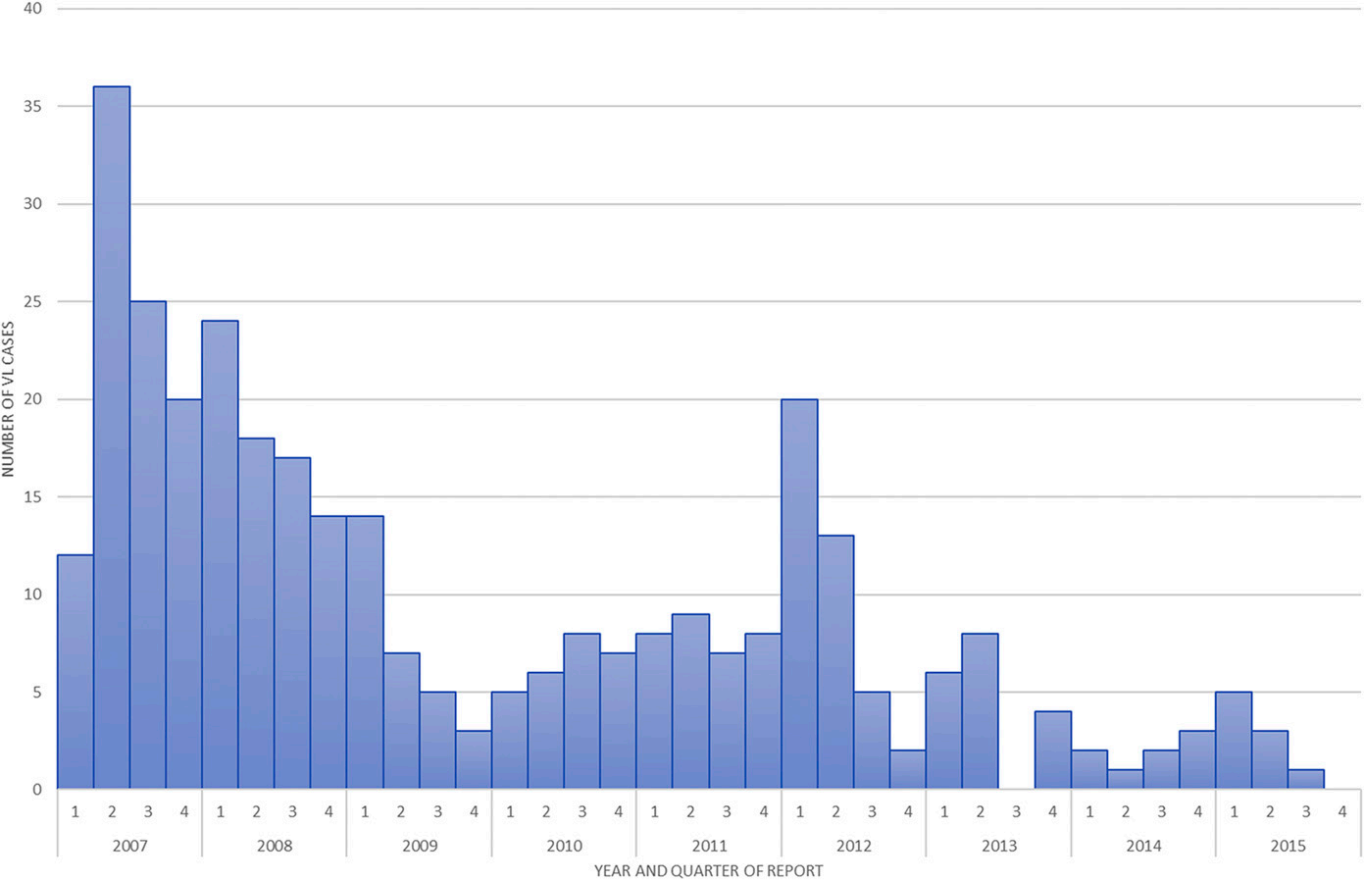
Incidence of visceral leishmaniasis (VL) in the Health and Demographic Surveillance Site area by quarter (2007–2015). This figure appears in color at www.ajtmh.org.

The epidemiological curve for the period 2007–2015 is shown in Figure 2. Over the years, there has been a sharp reduction in VL incidence as in other parts of Bihar.

### Data analysis.

To answer the question on which would be the optimal surface area for targeting the intervention measures, we calculated VL incidence rates within a number of a priori set perimeters around recent VL cases (reported in the previous 6-month period) and compared these with VL incidence rates among people living at more than 500 m distance from any of these recent VL cases (reference category). Henceforth we will refer to these recent VL patients as “index cases,” defined as “a person residing inside the study area diagnosed with VL during the previous 6-month period.”

With an average incubation period of 2–6 months and an average diagnostic delay in the order of 1–2 months, the bulk of secondary cases to arise from any VL case are expected in the next 6 months.^16^ For practical purposes, we assumed a biannual planning cycle, based on cases reported from January to June and from July to December. We realize that substantial numbers of secondary cases resulting from index cases early in the 6-month period are bound to be missed; nevertheless, this approach allows us to assess optimal buffer diameters for intervention measures during the next 6-month period. We created buffers of 0 (for same household contacts), 50, 75, 100, 200, 300, 400, and 500 m around all index cases occurring in the same 6-month period. We then determined the number (and proportion) of VL cases observed within those same buffer zones during the next 6-month period. We computed risk ratios for developing VL within the next 6 months between those living inside each of the seven buffer zones and those living at more than 500 m from any index case. To assess the feasibility of covering any of the proposed buffer diameters with intervention measures, we calculated the proportion of the total population that would have to be covered. We also explored the incremental yield and workload when expanding the size of buffer zones to be covered.

As a summary measure we calculated the total proportion of all cases reported over the period from July 1, 2007 till December 31, 2015 (*n* = 280, 48 cases reported during the first half of 2007 only figure as index cases) that arose within any of these buffer zones. We also calculated the average population size living within these boundaries. We then fitted a negative binomial model to compute an overall incidence rate ratio (IRR), always comparing those living within any of the seven buffer zones to those living at more than 500 m from any index case.

For each buffer segment we calculated a “cost/benefit” ratio for active screening based on the average 6-monthly VL incidence in that segment compared with the average 6-monthly VL incidence in the segment beyond 500 m from the nearest index cases. Although no actual costs were calculated, we assume that there is a fixed cost per person screened. The “cost/benefit ratio” is therefore defined as the number of persons needed to be screened to identify one new VL case.

Apart from the annual door-to-door surveys, the only control measure implemented was IRS based on dichlorodiphenyltrichloroethane (DDT). Implementation was very erratic and the effect of DDT on sandflies in Bihar has been shown to be minimal.^4,17^ We therefore did not control for any intervention measures in our analysis. Most VL cases recorded during the surveys had been diagnosed earlier. At the time of the survey, available records were verified and the date of diagnosis was recorded. Visceral leishmaniasis cases were also reported in between surveys through a network of village volunteers.

To account for the fact that the degree of clustering of cases may depend on the intensity of transmission, we included the 6-month period of report in our model as a random effect. To test for possible interactions, we regrouped the periods into two groups with contrasting incidence levels: 2007 till 2011 and 2012 till 2015.

**Figure 3.**
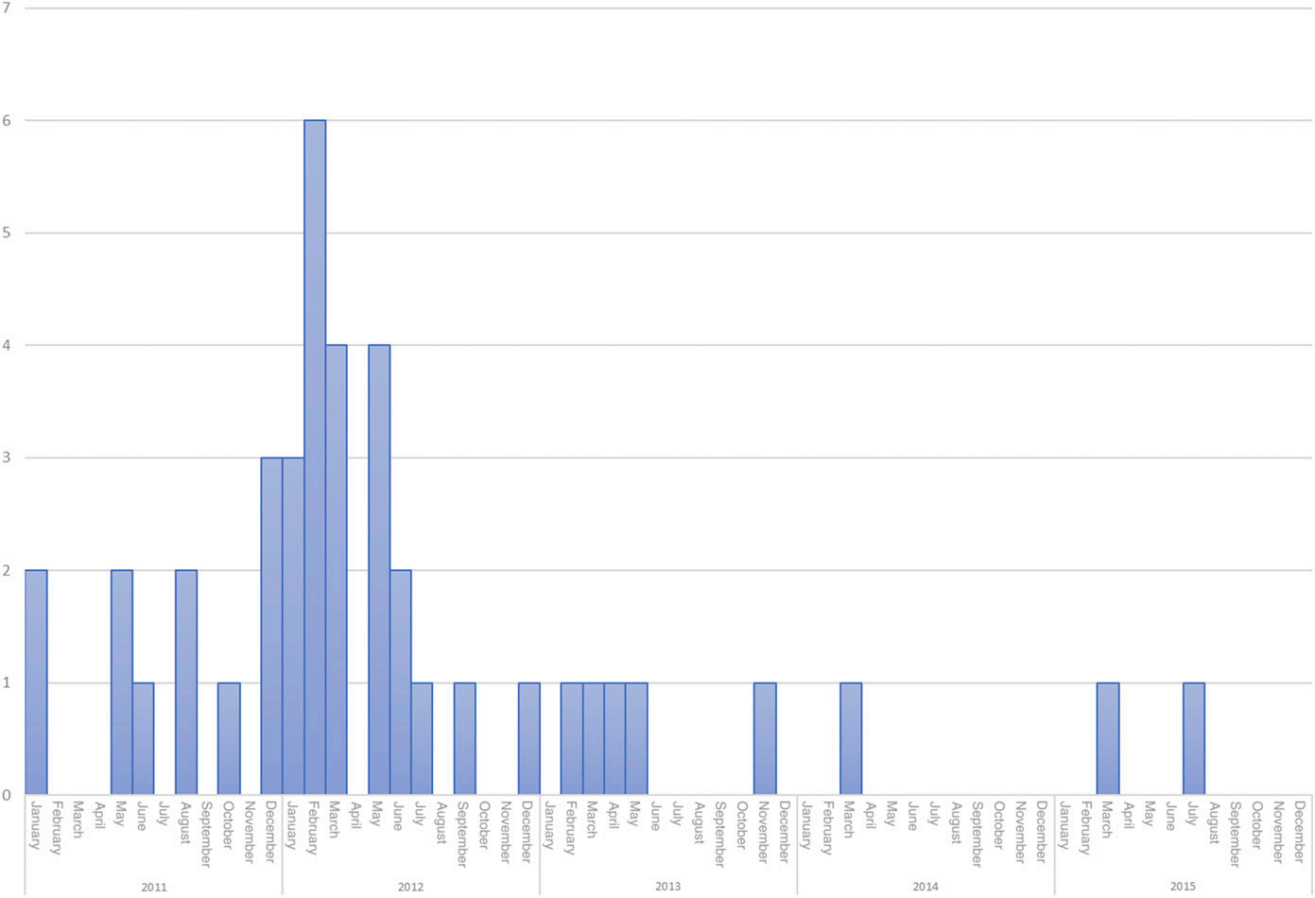
Epidemiological curve in one village that experienced an outbreak. This figure appears in color at www.ajtmh.org.

We did a more detailed analysis in one of the villages that experienced an outbreak that started in January 2011, and peaked in late 2011/first half 2012. The epidemiological curve observed in this village is shown in Figure 3. The question we tried to answer is whether the recommended buffer diameter of 500 m around the initial two cases diagnosed in January 2011 would have captured the households of VL cases arising during the remainder of that year and during the remainder of the outbreak. We also assessed whether a more limited IRS coverage might have been sufficient to capture all households eventually affected.

### Ethical considerations.

In this study, we used data from the Muzaffarpur HDSS. Ethical clearance for the data collection and analysis in the HDSS was obtained in an ethical review by the U.S. National Institutes of Health, as well as institutional review boards of the Institute of Medical Sciences, Banaras Hindu University, Varanasi, India, and the University of Iowa, Iowa. All subjects provided written informed consent; in case of illiterate subjects, a thumb print plus a signature of an independent witness were used. For minors under the age of 18, written informed consent was obtained from a parent or guardian. Informed consent procedures were approved by the respective review boards.

## Results

The distribution of VL cases was strongly clustered with high and statistically significant IRRs, showing a clear gradient in risk in function of distance from the index cases. When compared with those living at more than 500 m from any index case, the risk of being diagnosed with VL within the next 6-month period was on average 45.2, 15.4, 14.6, 13.4, 9.2, 7.1, 5.9, and 5.1 times higher for those living in the same household or within 50, 75, 100, 200, 300, 400, or 500 m, respectively. There was considerable variability by period (Supplemental Tables 1 and 2); however, the interaction between period (2007–2011 or 2012–2015) and buffer zone was not statistically significant. We therefore have no evidence in support of an increase or decrease of the degree of clustering over time. A summary of the main results is shown in Table 1.

**Table 1:** Frequency of VL cases and population living in the same household or within a 50, 75, 100, 200, 300, 400, or 500 m distance of recent (6 months earlier) VL index cases, and degree of clustering expressed by IRR compared with people living at more than 500 m distance

Perimeter (m)	Population (*n* [%])	VL cases (*n* [%])	IRR (95% CI)
Household	124 (0.1)	11 (3.9)	45.2 (23.8–85.6)
< 50	2,020 (2.2)	56 (20.0)	15.4 (9.8–24.2)
< 75	2,991 (3.3)	79 (28.2)	14.6 (10.1–21.2)
< 100	3,955 (4.3)	90 (32.1)	13.4 (8.5–21.3)
< 200	7,930 (8.6)	124 (44.3)	9.2 (5.6–15.1)
< 300	12,027 (13.1)	147 (52.5)	7.1 (4.6–11.2)
< 400	15,737 (17.1)	161 (57.5)	5.9 (4.0–8.7)
< 500	19,016 (20.7)	172 (61.4)	5.1 (3.4–7.5)
≥ 500	72,892 (79.3)	108 (38.6)	Ref.
Total	91,908 (100)	280 (100)	–

CI = confidence interval; IRR = incidence rate ratio; VL = visceral leishmaniasis.

The proportions of VL cases occurring within these perimeters ranged from 3.9% for the category “same household” to 61.4% within 500 m, although on average only 0.1% and 20.7% of the population live within these confines. Full details for each of the 6-month periods assessed are presented in Supplemental Tables 1–3.

As can be seen in Table 2, a circle with a 50-m radius drawn around the index households yields an additional 16% of VL cases, although only an additional 2% of the population needs to be covered. For the segment of 50–75 m the cost/benefit ratio is similar, beyond that it gets less favorable. However, only 28% of all VL cases lived within 75 m of an index case.

**Table 2:** Incremental yield in secondary VL cases identified when expanding the buffer diameter around index VL cases

Buffer diameter (m)	Additional VL cases found (%)	Average additional population to screen (% of total)	Cost benefit ratio of buffer segment (average number to screen per VL case detected)
Same household	11 (3.9)	124 (0.1)	124/11 = 11.3
1–49	45 (16.1)	1,896 (2.1)	1,896/45 = 42.1
50–74	23 (8.2)	971 (1.1)	971/23 = 42.2
75–99	11 (3.9)	964 (1.1)	964/11 = 87.6
100–199	34 (12.1)	3,975 (4.3)	3,975/34 = 116.9
200–299	23 (8.2)	4,097 (4.5)	4,097/23 = 178.1
300–399	14 (5.0)	3,710 (4.0)	3,710/14 = 265.0
400–499 m	11 (3.9)	3,279 (3.6)	3,279/11 = 298.1
≥ 500	108 (38.6)	72,892 (79.3)	72,892/108 = 674.9

VL = visceral leishmaniasis.

**Figure 4.**
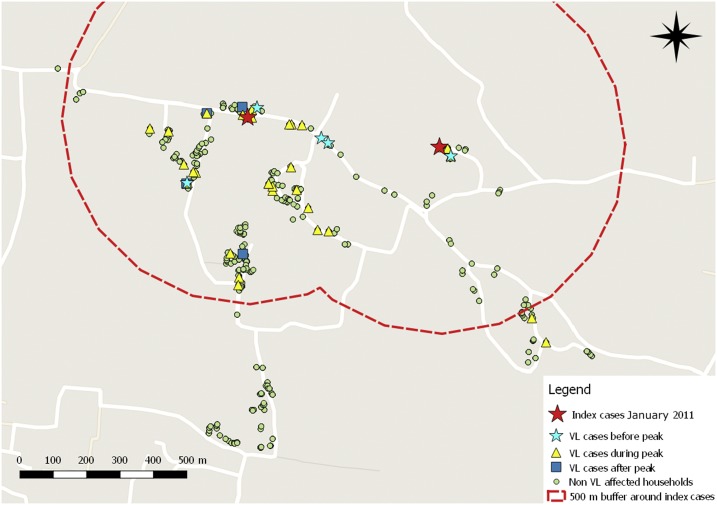
Spatial relation between visceral leishmaniasis (VL) cases with relation to the incidence peak (map created in QGIS version 2.14.19-Essen). This figure appears in color at www.ajtmh.org.

We explored in more detail the village that had experienced an outbreak, starting in the first quarter of 2011. Table 3 shows numbers and proportions of later cases in relation to distance from the initial two cases diagnosed in January 2011. All but two cases arose within a 500-m radius of those first two cases, the area to be targeted for IRS according to the guidelines (Figure 4). The two cases outside this perimeter did not occur until 2012.

**Table 3:** Visceral leishmaniasis cases in outbreak village after January 2011, population within different perimeters of initial two cases

Buffer diameter (m)	Cases after 2010 (%)	Population (%)	No. of households (%)
100	9 (23)	272 (13.0)	40 (12.8)
200	18 (46)	594 (28.5)	84 (26.8)
300	31 (79)	1,094 (52.5)	158 (50.5)
400	35 (90)	1,423 (68.2)	211 (67.4)
500	37 (95)	1,618 (77.6)	246 (78.6)
> 500	2 (5)	467 (22.4)	67 (21.4)

The exact locations of VL cases in relation to the main peak that was observed during the fourth quarter of 2011 and the first two quarters of 2012 are shown in Figure 4. Also shown is the 500 m buffer around the initial cases diagnosed in January 2011.

In Table 4, we show dates of diagnosis and distance to other cases for those that were diagnosed in the early phase of the outbreak (2011). Two cases were diagnosed in January, we assume they were infected in 2010. There were nine more cases during 2011, arising from May onward. As it becomes apparent from Table 4, when the radius of IRS coverage is reduced to 200 m, the households of five of nine later cases (cases 2, 3, 4, 6, and 9) would not be covered. A 300-m radius would still miss one case household (case 9), a 100-m radius would have missed six of nine (cases 2, 3, 4, 6, 7, and 9).

**Table 4:** Distance of secondary cases from initial cases assumed to have been infected in 2010

Date of diagnosis potential source case	Date of diagnosis secondary cases and distance in meters from potential source cases									
	Index 2	Case 1	Case 2	Case 3	Case 4	Case 5	Case 6	Case 7	Case 8	Case 9
	January 19	May 5	May 8	June 23	August 7	August 30	October 28	December 13	December 24	December 26
Index 1, January 12	522	36	206	240	227	556	213	177	11	429
Index 2, January 19	–	502	319	686	300	39	689	403	511	642

## Discussion

Our results corroborate previous findings that VL is strongly clustered at small area level, corresponding to what is known in India as a “tola,” a subdivision of the village. They also show that further clustering occurs at immediate neighborhood and household level. There is therefore a strong rationale to target control interventions to the close surroundings of reported VL cases. On average the risk for developing VL for someone living in the same household as a VL patient is 45 times higher than for someone living more than 500 m away. That risk gradually decreases to 10-fold and 5-fold for those living within 200 and 500 m, respectively, which is still a rather strong association. In our assessment, the incremental yield of extending interventions beyond the household of the VL case to a diameter of 50 m is very favorable; 16.1% of cases were found in this segment, although it makes up only 2.1 percent of the population. A further extension to 75 m is still favorable, yielding an additional 8.2% of cases versus 1.1% of the population. However, some caution is needed; because villages in Bihar are stretched out along roads, the increase in population to be covered when expanding the diameter around a case is mostly linear, not exponential as might be the case in other settings.

We acknowledge some limitations to our study. First, for the sake of the simulation, we assumed that transmission was strictly from index VL case to secondary VL case, making abstraction for example of post kala azar dermal leishmaniasis (PKDL) as a source of infection, and putative alternative reservoirs such as asymptomatic carriers. We allowed only for a single-time step, whereas VL cases can possibly remain infectious in the next sand fly season. PKDL cases could definitely remain a source of infection over longer periods, which would reduce the impact on transmission of IRS and active case finding around VL cases of the previous 6-month period. Seasonality also plays a role, as from December till February sand flies are virtually absent, so the impact and efficiency of interventions is in reality not constant throughout the periods studied.^18^ We also realize that even effective IRS within a specific buffer zone would not be sufficient to prevent all cases in the next 6-month period for several reasons. First of all IRS would not prevent VL in a person already infected but still in the incubation phase, secondly because people may also be infected in outdoor locations. Perry et al. found that during the warm months of the year, 95% of the population sleep outside.^19^ Nonetheless, although several of the aforementioned factors are important to explain maintenance of transmission, we believe our method provides a practical way of comparing the potential yield of different interventions.

Other considerations may apply for IRS than for active case finding. Effective IRS could contain an outbreak in its early stages, provided the net is cast wide enough. Overall, we saw that roughly 60% of VL cases occur within 500 m of cases reported during the previous 6 months, the remainder are further away. In a more detailed analysis of one village that experienced a typical outbreak, we saw that effective IRS in a 500-m perimeter around the two initial cases might have stopped the outbreak in its early stages. Based on the observations in this village, reducing this diameter does not seem advisable, as substantial proportions of households in which cases later occurred were at more than 200-m distance from the initial case households. Even if the flight range of sand flies is limited, we did observe VL cases emerging within a short timespan yet hundreds of meters apart. However more such observations are required before findings can be generalized.

## Conclusion

This observational study on a large population cohort confirms the rationale of the present guidelines to conduct IRS in a perimeter of 500 m around index cases or covering the entire village. The one outbreak studied in detail would in all probability have been contained by effective IRS in a perimeter of 500 m around the early VL cases. For reactive case finding it is highly recommended to not only examine household contacts, but also those residing within 50–75 m around the affected household as mentioned by Huda et al.^10^

## Supplementary Material

Supplemental Tables
