# Supplementary material for: Visceral Leishmaniasis in the Muzaffapur Demographic Surveillance Site: A Spatiotemporal Analysis
Source: Am J Trop Med Hyg. 2018 Oct 8;99(6):1555–61. doi: 10.4269/ajtmh.18-0448 (PMC6283495; doi:10.4269/ajtmh.18-0448)
Supplement: Supplementary file 1 [file tpmd180448.SD1.pdf]

Supplemental Table 1: Number of VL cases reported within six months after the end of the quarter of report of index cases, by quarter of report of index case and distance from index case

Year and quarter of index cases	Number of VL cases (% of total)								
	Total	Same household	Within 50m	Within 75m	Within 100m	Within 200m	Within 300m	Within 400m	Within 500m
2007, q1+2	45	3 (7)	12 (27)	14 (31)	14 (31)	24 (53)	28 (62)	29 (64)	32 (71)
2007, q3+4	42	3 (7)	10 (24)	18 (43)	19 (45)	22 (52)	25 (60)	29 (69)	30 (71)
2008, q1+2	31	1 (3)	6 (19)	8 (26)	13 (42)	15 (48)	22 (71)	24 (77)	26 (84)
2008, q3+4	21	0 (0)	7 (33)	7 (33)	7 (33)	9 (43)	10 (48)	12 (57)	12 (57)
2009, q1+2	8	0 (0)	0 (0)	2 (25)	2 (25)	2 (25)	2 (25)	2 (25)	2 (25)
2009, q3+4	11	1 (9)	1 (9)	1 (9)	1 (9)	2 (18)	2 (18)	3 (27)	3 (27)
2010, q1+2	15	0 (0)	0 (0)	2 (13)	2 (13)	3 (20)	4 (27)	5 (33)	5 (33)
2010, q3+4	17	0 (0)	2 (12)	2 (12)	2 (12)	2 (12)	3 (18)	5 (29)	5 (29)
2011, q1+2	15	1 (7)	5 (33)	5 (33)	6 (40)	8 (53)	9 (60)	9 (60)	10 (67)
2011, q3+4	33	0 (0)	6 (18)	9 (27)	12 (36)	20 (61)	22 (67)	22 (67)	26 (79)
2012, q1+2	7	1 (14)	4 (57)	4 (57)	4 (57)	5 (71)	6 (86)	6 (86)	6 (86)
2012, q3+4	14	0 (0)	1 (7)	3 (21)	4 (29)	6 (43)	6 (43)	6 (43)	6 (43)
2013, q1+2	4	0 (0)	1 (25)	1 (25)	1 (25)	1 (25)	2 (50)	2 (50)	2 (50)
2013, q3+4	3	0 (0)	0 (0)	1 (33)	1 (33)	1 (33)	1 (33)	2 (67)	2 (67)
2014, q1+2	5	0 (0)	0 (0)	0 (0)	0 (0)	0 (0)	0 (0)	0 (0)	0 (0)
2014, q3+4	8	1 (13)	1 (13)	1 (13)	2 (25)	4 (50)	4 (50)	4 (50)	4 (50)
2015, q1+2	1	0 (0)	0 (0)	0 (0)	0 (0)	0 (0)	1 (100)	1 (100)	1 (100)
TOTAL	280[illegible]	11 (3.9)	56 (20.0)	78 (27.9)	90 (32.1)	124 (44.3)	147 (52.5)	161 (57.5)	172 (61.4)
