# Supplementary material for: Visceral Leishmaniasis in the Muzaffapur Demographic Surveillance Site: A Spatiotemporal Analysis
Source: Am J Trop Med Hyg. 2018 Oct 8;99(6):1555–61. doi: 10.4269/ajtmh.18-0448 (PMC6283495; doi:10.4269/ajtmh.18-0448)
Supplement: Supplementary file 2 [file tpmd180448.SD2.pdf]

Supplemental Table 2: Risk ratios of developing VL within a 6 months period after the end of the quarter of report of index cases, by distance from index case with those living at more than 500 meters as reference category

Year and quarter of index cases	Distance from index case							
	Same household	Within 50m	Within 75m	Within 100m	Within 200m	Within 300m	Within 400m	Within 500m
2007, q1+2	41(12-144)	11(5.0-24)	8.9(4.2-19)	6.6(3.1-14)	6.0(3.1-12)	5.0(2.6-9.6)	4.2(2.2-8.0)	3.8(2.0-7.2)
2007, q3+4	38(11-134)	9.2 (4.0-21)	12(5.8-25)	9.8(4.8-20)	5.9(2.9-12)	4.6(2.3-9.1)	4.0(2.0-7.8)	3.4(1.7-6.7)
2008, q1+2	49(5.8-422)	16(4.9-52)	15(4.9-45)	18 (6.3-50)	11(3.9-29)	11(4.0-28)	9.4(3.6-25)	9.0(3.5-24)
2008, q3+4	NA	19(7.2-52)	13(4.9-35)	9.8(3.6-26)	6.9(2.7-17)	5.2(2.1-13)	5.1(2.1-12)	4.3(1.8-10)
2009, q1+2	NA	NA	7.0(1.4-35)	5.8(1.2-29)	5.0(1.0-25)	3.0(0.6-15)	1.9(0.4-9.3)	1.1(0.2-5.5)
2009, q3+4	156(20-1232)	6.8(0.8-54)	4.4(0.5-35)	3.3(0.4-26)	3.3(0.7-15)	2.0(0.4-9.2)	1.9(0.5-7.3)	1.5(0.4-5.8)
2010, q1+2	NA	NA	6.0(1.3-28)	4.8(1.1-22)	4.0(1.1-14)	3.9(1.2-12)	4.0(1.4-12)	3.5(1.2-10)
2010, q3+4	NA	5.7(1.3-26)	3.7(0.8-17)	2.8(0.6-12)	1.2(0.3-5.3)	1.1(0.3-1.8)	1.3(0.5-3.8)	1.1(0.4-3.1)
2011, q1+2	159(19-1345)	45(13-157)	39(12-126)	30(9.3-99)	18(5.8-54)	12(3.9-34)	8.5(2.9-25)	7.7(2.6-23)
2011, q3+4	NA	39(13-117)	36(13-96)	35(14-89)	25(10-58)	18(7.7-42)	15(6.4-32)	15(6.3-33)
2012, q1+2	332(21-5294)	128(14-1140)	91(10-816)	76(8.5-679)	50(5.9-429)	38(4.6-318)	27(3.2-222)	22(2.6-181)
2012, q3+4	NA	16(2.0-129)	31(8.3-118)	34(10-112)	29(10-84)	17(5.9-49)	13(4.5-38)	10(3.6-30)
2013, q1+2	NA	22(2.0-241)	15(1.4-167)	11(1.0-124)	5.5(0.5-60)	6.3(0.9-45)	4.3(0.6-31)	3.2(0.5-23)
2013, q3+4	NA	NA	78(4.8-1240)	51(3.2-809)	20(1.3-325)	14(0.9-227)	22(2.0-244)	18(1.6-200)
2014, q1+2	NA	NA	NA	NA	NA	NA	NA	NA
2014, q3+4	686(79-5967)	38(4.3-340)	23(2.6-204)	34(6.1-183)	26(6.4-103)	18(4.5-71)	15(3.6-58)	12(3.1-50)
2015, q1+2	NA	NA	NA	NA	NA	NA	NA	NA
