# Supplementary material for: Visceral Leishmaniasis in the Muzaffapur Demographic Surveillance Site: A Spatiotemporal Analysis
Source: Am J Trop Med Hyg. 2018 Oct 8;99(6):1555–61. doi: 10.4269/ajtmh.18-0448 (PMC6283495; doi:10.4269/ajtmh.18-0448)
Supplement: Supplementary file 3 [file tpmd180448.SD3.pdf]

Supplemental Table 3: Proportion of the population to be covered in function of buffer diameter around index cases (total population 91,908)

Year and quarter of index cases	Buffer diameter/ no. of persons living within (% of total population)							
	Case house-holds only	Within 50m	Within 75m	Within 100m	Within 200m	Within 300m	Within 400m	Within 500m
2007, q1+2	311(0.3)	4,760(5.1)	6,729(7.3)	9,121(9.9)	17,078(18.6)	24,096(26.2)	29,900(32.5)	36,137(39.3)
2007, q3+4	348(0.4)	4,816(5.2)	6,649(7.2)	8,551(9.3)	16,554(18.0)	24,210(26.4)	32.336(35.2)	38,853(42.3)
2008, q1+2	236(0.3)	4,372(4.8)	6,287(6.8)	8,533(9.3)	16,471(17.9)	23,914(26.0)	29,934(32.6)	33,541(36.5)
2008, q3+4	204(0.2)	2,838 (3.2)	4,133(4.5)	5,588(6.1)	10,231(11.1)	15,012(16.3)	18,472(20.1)	21,609(23.5)
2009, q1+2	114(0.1)	2,203(2.4)	3,375(3.7)	4,068(4.4)	7,745(8.4)	12,581(13.7)	17,037(18.5)	21,204(23.1)
2009, q3+4	59 (0.06)	1,365(1.5)	2,102(2.3)	2,819(3.1)	5,659(6.2)	9,452(10.3)	14,326(15.6)	18,053(19.6)
2010, q1+2	61 (0.07)	1,750 (1.9)	2,669(2.9)	3,320(3.6)	6,057 (6.6)	8,228(9.0)	10,181(11.1)	11,373(12.4)
2010, q3+4	113(0.1)	1,929(2.1)	2,988(3.3)	4,007(4.4)	9,330(10.2)	15,441(16.8)	20,836(22.7)	25,479(27.7)
2011, q1+2	92 (0.1)	1,608(1.8)	2,267(2.5)	2,890(3.1)	6,613(7.2)	11,423(12.4)	15,337(16.7)	18,897(20.6)
2011, q3+4	90 (0.1)	1,593(1.7)	2,619(2.9)	3,568(3.9)	8,492(9.2)	12,965(13.8)	15,270(16.6)	18,723(20.4)
2012, q1+2	217(0.2)	2,261(2.5)	3,158(3.4)	3,796(4.1)	7,185(7.8)	11,311(12.3)	16,176(17.6)	19,813(21.6)
2012, q3+4	48(0.05)	664(0.7)	1,023(1.1)	1,266(1.4)	2,209(2.4)	3,796(4.1)	4,934(5.4)	6,208(6.8)
2013, q1+2	79 (0.09)	1,605(1.8)	2,312(2.5)	3,123(3.4)	6,435(7.0)	11,073(12.1)	16,142(17.6)	21,729(23.6)
2013, q3+4	26(0.03)	559(0.6)	1,067(1.2)	1,635(1.8)	4,070(4.4)	5,834(6.4)	7,476(8.1)	9,122(9.9)
2014, q1+2	23(0.03)	625(0.7)	1,041 (1.1)	1,516(1.7)	3,110(3.4)	4,589(5.0)	5,447(5.9)	5,976(6.5)
2014, q3+4	31 (0.03)	559(0.6)	930(1.0	1,269(1.4)	3,313(3.6)	4,762(5.2)	5,858(6.4)	6,835(7.4)
2015, q1+2	48(0.05)	892(1.0)	1,504(1.6)	2,164(2.4)	4,255(4.6)	6,038(6.6)	7,835(8.5)	9,713(10.6)
